# Kinematical Effects of a Mandibular Advancement Occlusal Splint on Running until Exhaustion at Severe Intensity

**DOI:** 10.3390/s24186032

**Published:** 2024-09-18

**Authors:** Filipa Cardoso, Mário J. Costa, Manoel Rios, João Paulo Vilas-Boas, João Carlos Pinho, David B. Pyne, Ricardo J. Fernandes

**Affiliations:** 1 Centre of Research, Education, Innovation and Intervention in Sport, CIFI2D, and Porto Biomechanics Laboratory, LABIOMEP-UP, Faculty of Sport, University of Porto, 4200-450 Porto, Portugal; mjcosta@fade.up.pt (M.J.C.); or manoel.rios@hotmail.com (M.R.); jpvb@fade.up.pt (J.P.V.-B.); ricfer@fade.up.pt (R.J.F.); 2 Faculty of Dental Medicine, University of Porto, 4200-393 Porto, Portugal; pinhojc53@gmail.com; 3 Institute of Science and Innovation in Mechanical and Industrial Engineering, INEGI, Faculty of Engineering, University of Porto, 4200-465 Porto, Portugal; 4 Research Institute for Sport & Exercise, University of Canberra, Canberra 2617, Australia; david.pyne@canberra.edu.au

**Keywords:** occlusal splints, mandibular repositioning, velocity at maximal oxygen uptake, running biomechanics, linear kinematics, angular kinematics, time to exhaustion, running economy

## Abstract

The effects of occlusal splints on sport performance have already been studied, although their biomechanical impacts are often overlooked. We investigated the kinematical changes during running until exhaustion at severe intensity while wearing a mandibular advancement occlusal splint. Twelve trained runners completed (i) an incremental protocol on a track to determine their velocity corresponding to maximal oxygen uptake and (ii) two trials of square wave transition exercises at their velocity corresponding to maximal oxygen until exhaustion, wearing two occlusal splints (without and with mandibular advancement). Running kinematics were compared within laps performed during the square wave transition exercises and between splint conditions. The mandibular advancement occlusal splint increased the running distance covered (~1663 ± 402 vs. 1540 ± 397 m, *p* = 0.03), along with a noticeable lap effect in decreasing stride frequency (*p* = 0.04) and increasing stride length (*p* = 0.03) and duty factor (*p <* 0.001). No spatiotemporal differences were observed between splints, except for improved balance foot contact times in the mandibular advancement condition. An increased knee flexion angle at initial contact (*p* = 0.017) was noted along laps in the non-advancement condition, despite the fact that no differences between splints were found. Running patterns mainly shifted within laps rather than between conditions, indicating that a mandibular advancement occlusal splint had a trivial kinematical effect.

## 1. Introduction

Biomechanical changes often occur as runners progress over time [1,2,3], with fatigue being one of the primary factors contributing to the substantial running modifications on lower limb mechanics [4,5,6]. Therefore, runners adopt different and individual strategies in trying to compensate for exhaustion, thereby making them effective in maintaining the same force output as they approach exhaustion [5,7,8]. Since fatigue can play a major role in modifying running patterns, the kinematic adjustments may be even more impactful when running at a severe exercise intensity, where exhaustion is reached within a short time period, typically between 3 and 10 min [9,10,11].

Mandibular advancement occlusal splints are extensively recognized as an alternative treatment for obstructive sleep apnea syndrome given their positive effects in increasing the upper airway size and enhancing airflow [12]. Based on these ventilatory effects, some studies have also investigated their use regarding potential benefits during exercise [13,14,15]. Through necessary design and manufacturing modifications, mandibular advancement devices have demonstrated positive physiological effects and sport performance improvements, particularly increased ventilation and prolonged exercise time to exhaustion [13,15]. If mandibular advancement positively influences time to exhaustion, runners could eventually sustain exercise for extended periods with fewer or more minor kinematical changes. However, it remains unclear whether the kinematic changes observed with the use of a specific occlusal splint are a consequence of the improved physiological variables, or whether they are due to a mechanism that affects gait patterns as a direct result of manipulated mandibular position.

Although research on the impact of mandibular advancement occlusal splints on sport performance is limited, the effects on gas exchange have garnered significant attention due to their direct influence on the upper airway. Furthermore, small adjustments in mandibular position have also been linked to changes in gait and running patterns [16,17,18], prompting inquiry into whether these devices may also affect exercise biomechanics. While indirect evidence indicates that changes in mandibular position might affect body posture [17,19], the extent to which such alterations influence gait patterns, particularly during exercise activities with a broader range of motion than standing or walking, remains uncertain. This study aimed to analyze the kinematical effects of a mandibular advancement occlusal splint during running at severe intensity, hypothesizing that runners (i) would alter their running technique as they approach exhaustion and (ii) would extend the distance covered, and, eventually, influence running patterns during a running until exhaustion at severe intensity.

## 2. Materials and Methods

### 2.1. Participants

Twelve trained male runners (26.3 ± 4.4 years old, 176.0 ± 6.3 cm of height, 65.5 ± 7.2 kg of body mass, 13 ± 5 years of running experience, and 12 ± 4 h of weekly training) volunteered to participate in the current study. All included subjects were (i) over 18 years of age; (ii) without severe dental and/or periodontal disease, and/or temporomandibular joint disorders; (iii) not currently under orthodontic treatment; and (iv) not suffering from any restricting injury within the three months prior to the data collection. All participants provided written informed consent upon receiving detailed information on the study’s aims and procedures and could withdraw from the study at any time. The study was conducted in accordance with the Declaration of Helsinki on human experimentation and was approved by the local ethics board.

### 2.2. Experimental Procedures

**Figure 1:**
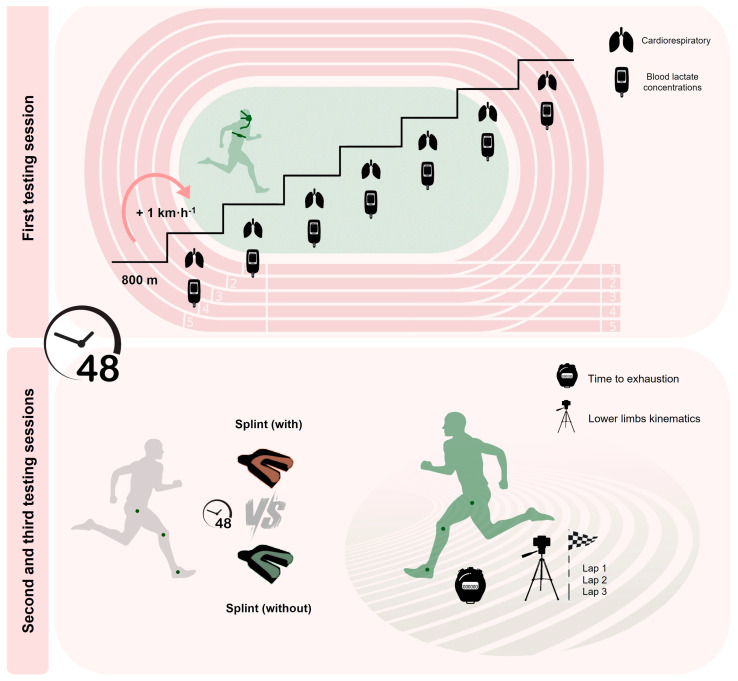
Implemented setup for the three experimental sessions.

Each participant completed three experimental sessions on a 400 m outdoor track field, with a 48 h interval between each session. To minimize circadian variations, subjects were tested at the same time of the day (±2 h), with wind conditions maintained below 2 m·s*^−^*^1^ (assessed by a digital handheld anemometer, Mastech*^®^* MS6252A, Charlotte, NC, USA). All participants were advised to avoid strenuous exercise the day before the experiments and to maintain usual training routines. During the first session, participants completed a running intermittent incremental protocol of 800 m steps (with increments of 1 km·h*^−^*^1^ per step and 30 s intervals in between) until volitional exhaustion, to assess the maximal oxygen uptake ($\overset{˙}{V}$O_2max_) and associated velocity (v$\overset{˙}{V}$O_2max_) [20,21]. Afterwards, the participants performed, in a randomized and counterbalanced order, two trials of square wave transition exercises at the predetermined v$\overset{˙}{V}$O_2max_ until exhaustion [21,22] wearing an intraoral occlusal splint without and with mandibular advancement (Figure 1). The intraoral occlusal splints were custom manufactured beforehand for each participant by a specialized dentist following specific fabrication procedures [14]. The splints without mandibular advancement were designed so as not to alter mandibular position and were carefully trimmed on the occlusal surfaces to avoid any interference with the runners’ dental occlusion or occlusal vertical dimension.

**Figure 2:**
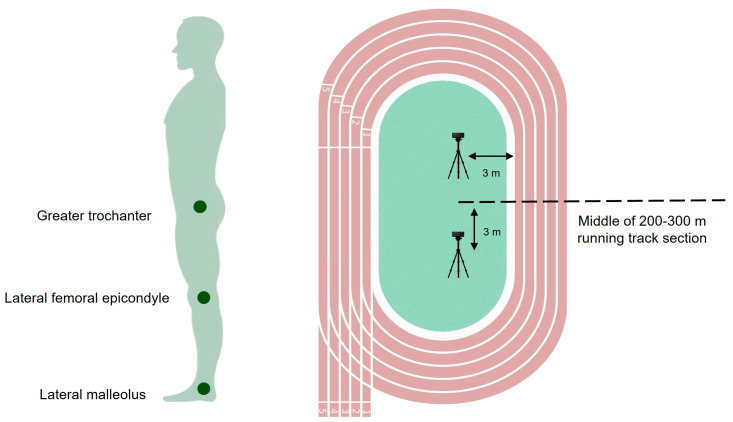
Landmarks and camera-specific positions adopted for the square wave transition exercise trials.

During the incremental protocol and square wave transition exercises, $\overset{˙}{V}$O_2max_ was continuously measured using a portable telemetric gas analysis system (K4b2, Cosmed, Rome, Italy) fixed to the runner′s back, and capillary blood samples were collected from the fingertip (5 µL, Lactate Pro2; Arkay, Inc., Kyoto, Japan) for posterior lactate concentration analysis [19]. Blood lactate concentrations were measured at rest, during each 30 s interval, immediately at the end of the exercise, and at the 3rd min post-exercise cessation [14]. Capillary blood collection was always performed by applying controlled pressure to the finger to minimize volume variations and ensure consistent results, and all initial blood samples were discarded to eliminate contaminants and guarantee measurement accuracy. For the square wave transition exercise trials, subjects were marked manually with black skin landmarks on the greater trochanter, lateral femoral epicondyle, and lateral malleolus (Figure 2) [22]. The time sustained at v$\overset{˙}{V}$O_2max_ was obtained using a stopwatch (Seiko, Tokyo, Japan) and biomechanical assessment was conducted using two high-definition cameras (GoPro HERO6 Black, San Mateo, CA, USA), operating at 120 Hz and strategically positioned 3 m from the middle of the 200–300 m running track section and 3 m from the 1st lane (Figure 2) [14].

### 2.3. Data Analysis

From the running incremental protocol, $\overset{˙}{V}$O_2max_ was considered when a $\overset{˙}{V}$O_2_ plateau (<2.1 mL·kg^−1^·min^−1^) was observed despite an increase in running velocity, a respiratory exchange ratio >1.1, blood lactate concentrations >8 mm·L*^−^*^1^, heart rate >90% of [220-age] and volitional exhaustion (controlled through visual inspection and individual case analysis), and v$\overset{˙}{V}$O_2max_ computed as the running velocity of the first incremental step that elicited $\overset{˙}{V}$O_2max_ [9,20]. All spatiotemporal and angular kinematic variables were assessed in Kinovea*^®^* software (v. 0.9.5, Boston, MA, USA) from the first, penultimate, and ultimate laps (defined as lap 1, 2, and 3, respectively) performed during the square wave transition exercise trials. For each runner, three running cycles (strides) per lap were analyzed for the respective splint conditions.

**Figure 3:**
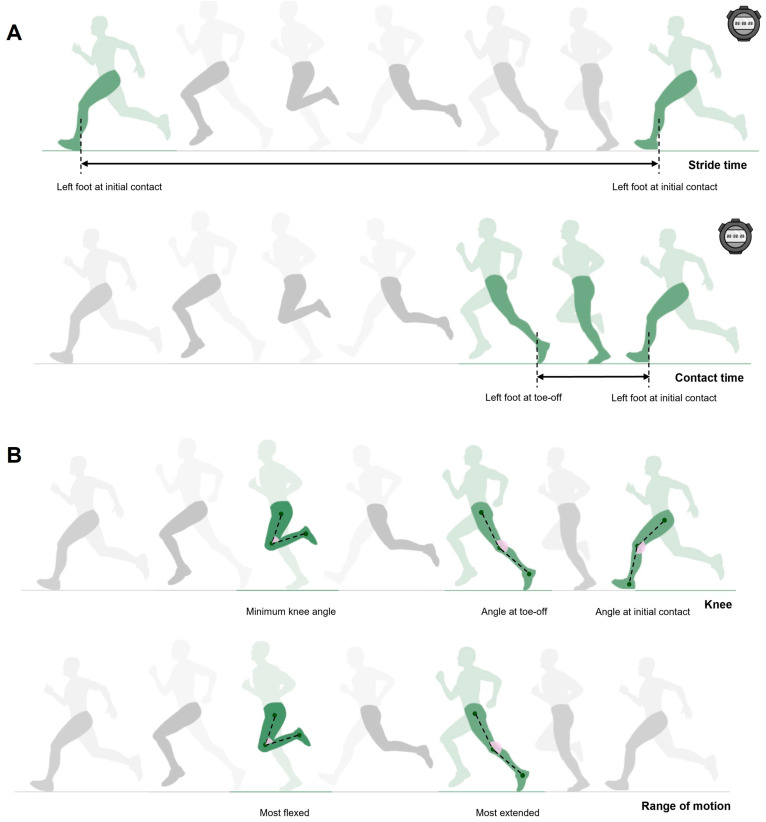
Running temporal and knee angular variables (panels (**A**,**B**), respectively) analyzed in Kinovea software while running until exhaustion at severe intensity.

The total distance covered, stride frequency, stride length, and duty factor during running until exhaustion at severe intensity were calculated based on the following equations [23,24,25]: (i) total distance (m) = v$\overset{˙}{V}$O_2max_ (m·s^−1^)· time sustained at v$\overset{˙}{V}$O_2max_ (s); (ii) stride frequency (Hz) = stride time (s)^−1^; and (iii) stride length (m) = v$\overset{˙}{V}$O_2max_ (m·s^−1^) · stride frequency (Hz)^−1^ and (iv) duty factor = contact time (s) · stride time (s)^−1^, where stride time (s) is the duration to complete a running cycle, i.e., the time between successive initial contacts of the same foot and contact time is the time from initial contact to toe-off of the same foot (Figure 3, panel A). All angular kinematic analyses were conducted exclusively in the left sagittal plane, where (i) knee angle was measured at initial contact and toe-off moments; (ii) the minimum knee angle was assessed at maximum knee flexion during the running cycle; and (iii) the knee range of motion was calculated as the difference between the most extended and most flexed knee positions during the whole running cycle (Figure 3, panel B) [26,27,28]. All spatiotemporal and angular variables were analyzed and measured by the same operator.

### 2.4. Statistical Analysis

Assuming biomechanical changes within laps with a moderate effect size (f = 0.25), an α = 0.05, a power of 0.8, and a correlation among repeated measures of 0.8, the sample size calculation resulted in the recruitment of 10 participants (G*Power, v. 3.1.9.7, Düsseldorf, Germany). The effect of the lap and splint and the interaction effect of lap*splint on biomechanical variables were investigated using two-way repeated measures ANOVA. Sphericity was assessed using the Mauchly test and, if violated, the Greenhouse–Geisser correction was applied. In the event of a significant main effect, a Bonferroni post hoc multiple comparison was conducted. Comparisons between splints for the distance covered and differences between feet were undertaken using paired t-tests. For each ANOVA and paired t-test, partial eta-squared (η²) and Cohen’s *d* (*d*) were computed as a measure of effect size (respectively). All data were reported as mean ± standard deviation and the statistical analyses were performed on SPSS (v. 29.0, IBM Corp., Armonk, NY, USA) with the significance level set at *p* ≤ 0.05.

## 3. Results

**Figure 4:**
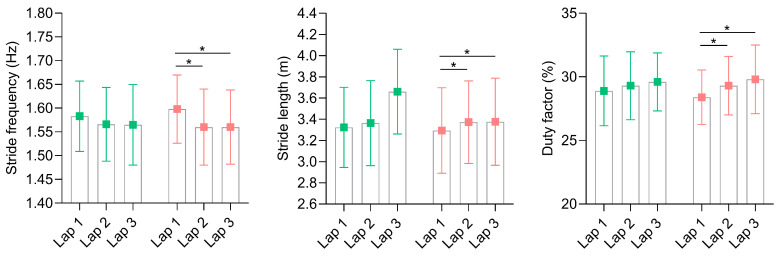
Running spatiotemporal variables during running until exhaustion at severe intensity for both tested occlusal splints (green and red for conditions without and with mandibular advancement, respectively). * *p* ≤ 0.05 indicates differences within laps.

The distance covered by the runners ranged from 1200 to 2329 m and from 1223 to 2400 m for the occlusal splints without and with mandibular advancement (respectively), with the condition involving mandibular advancement resulting in runners covering ~8% more distance (~1663 ± 402 vs. 1540 ± 397 m, *p* = 0.03, *d* = 0.62). The spatiotemporal variables during running until exhaustion at severe intensity while wearing both occlusal splints are depicted in Figure 4. A significant main effect of lap was identified on stride frequency and stride length (*p* = 0.04 and 0.03; η^2^ = 0.32 and 0.35, respectively) in the mandibular advancement condition, with post hoc analysis showing a decreased stride frequency between the first and second laps, and between the first and third laps (*p* = 0.02 and 0.05, respectively), and an increased stride length between the first and second laps, and between the first and third laps (*p* = 0.01 and 0.03, respectively). Nevertheless, there were no main effects of splint nor interaction lap*splint on the same variables.

**Figure 5:**
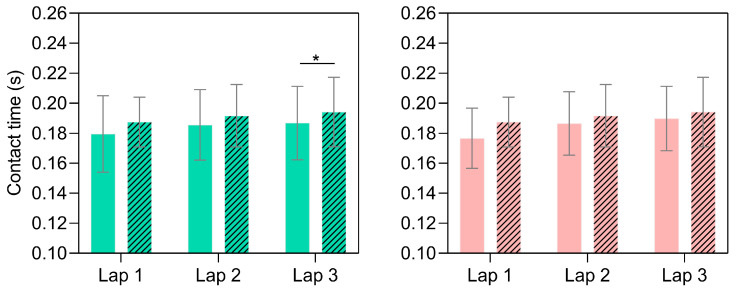
Contact time for each foot (solid and striped for right and left foot, respectively) during running until exhaustion at severe intensity for both tested occlusal splints (green and red for conditions without and with mandibular advancement, respectively. * *p* ≤ 0.05 indicates differences between feet.

The duty factor during running until exhaustion at severe intensity showed a main effect of a lap for the condition with mandibular advancement (*p* ≤ 0.001, η^2^ = 0.52, Figure 4), with post hoc comparisons revealing an increased duty factor from the first to second lap and from the first to third lap (*p* = 0.009 and 0.01, respectively). Figure 5 illustrates the contact time performed for each foot across the different laps and splint conditions tested. No considerable differences in contact times between the right and left feet during the first and second laps were observed for the non-advancement condition, despite the fact that the left foot showed increased contact time compared to the right foot in the third lap (~4%, *p* = 0.03, *d* = 0.74). However, no differences between feet were observed in the condition with mandibular advancement within each lap analyzed.

**Figure 6:**
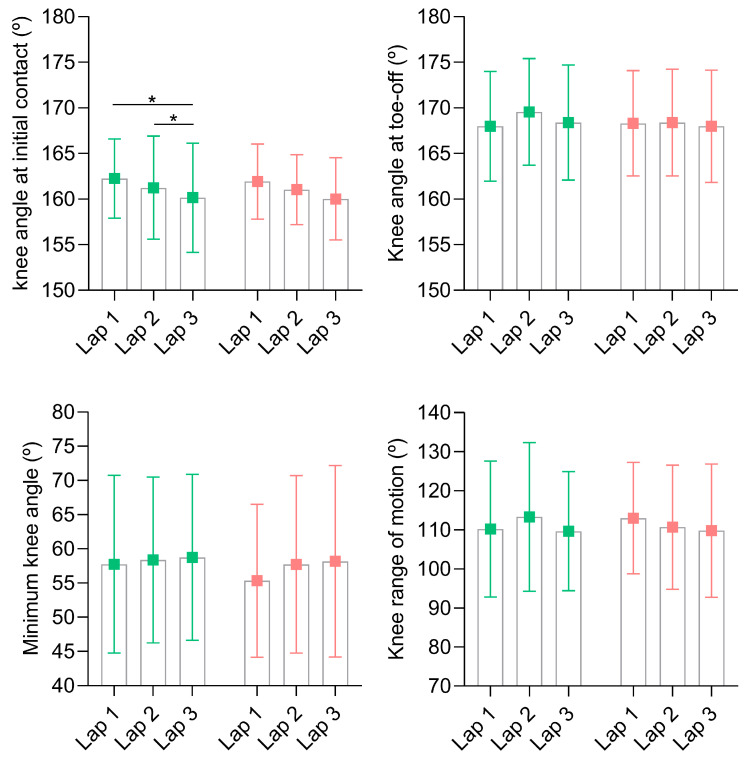
Knee angular kinematics during running until exhaustion at severe intensity for both tested occlusal splints (green and red for conditions without and with mandibular advancement, respectively). * *p* ≤ 0.05 indicates differences within laps.

A significant main effect of lap was identified for the knee at initial contact in the condition without mandibular advancement (*p* = 0.017; η^2^ = 0.37, Figure 6), with a decreased knee angle at initial contact between the first and third laps and between the second and third laps *(p* = 0.049 and 0.042, respectively). At toe-off, no main effects were evident within laps or between splint conditions nor within the interaction lap*splint for the knee angle values. Regarding the minimum knee angle, there was a main effect for lap (*p* = 0.01; η^2^ = 0.33) in the mandibular advancement condition, although pairwise comparisons did not indicate differences between laps. Moreover, no splint or lap*splint interaction effects were observed for the minimum knee angle and no main effects were evident within laps, between splint conditions, or within an interaction lap*splint for the knee range of motion.

## 4. Discussion

Our results indicate that running patterns change as runners approach exhaustion at severe intensity, demonstrating that these mechanical adaptations help sustain exercise despite progressive fatigue levels [1,29,30]. Moreover, even though only trivial kinematical changes were observed when a mandibular advancement occlusal splint was worn, runners were able to cover more distance under this experimental condition. Although the topic remains controversial on how mandibular position manipulations may interfere with postural control and head position, consequently affecting running patterns [17,18], small kinematical changes may be indirectly linked to the proven physiological effects of wearing specific occlusal splints during exercise. This is particularly evident for mandibular advancement occlusal splints, which have shown biomechanical modifications alongside ergogenic effects on ventilation during running at different exercise intensities [13,16].

While changes in spatiotemporal variables under fatiguing states have been previously described [31,32,33], other studies reported no changes in either stride frequency or stride length [34]. These findings corroborate our data concerning the consistent stability of the spatiotemporal variables throughout the running until exhaustion at severe intensity trials. Interestingly, the lack of a splint effect for linear kinematic variables indicates that neither the non-advancement nor the advancement conditions markedly influenced them, as confirmed by previous results [14]. Nevertheless, a mandibular advancement occlusal splint can elicit increases in step frequency and decreases in stride length during a treadmill running incremental protocol until exhaustion [13,16].

The rising duty factor observed across laps within the mandibular advancement condition indicates a greater proportion of the running cycle time spent on the ground, which is consistent with previous research on running until exhaustion around and at v$\overset{˙}{V}$O_2max_, where duty factor has been shown to increase with fatigue [34]. Indeed, an increased duty factor during a fatigued state is seen as a protective strategy to reduce the load on the musculoskeletal system, including impact shock [34]. Based on our data, we also observed that splint conditions had little effect on the duty factor within laps, and given the lack of relevant literature on the influence of intraoral occlusal splints on this specific variable, further comparative analysis is currently limited.

The non-advancement splint increased the difference between the left and the right foot contact times more than the mandibular advancement condition, aligning with previously reported findings that indicated that specific mandibular repositioning appliances might enhance step symmetry during running [16,17]. While slightly more balanced contact times between feet were observed in the mandibular advancement occlusal splint, these changes were small and likely of little practical importance. Nevertheless, as running asymmetry is deemed important for both injury risk and running economy [17,35], future studies should investigate how personalized adjustments in running patterns facilitated by mandibular advancement splints can contribute to these factors.

Regarding knee angular kinematics, a lap effect was observed only in the non-advancement condition, indicating increased knee flexion at initial contact throughout the laps performed. A more flexed knee position at initial contact has been reported previously under fatigue conditions, with the likely function of helping to manage energy cost and absorb impact forces when muscles are exhausted [1,28,36]. Although our data did not reveal differences in knee angular kinematics between experimental conditions, the use of mandibular advancement splints has been shown to reduce peak knee flexion and knee range of motion at specific stages during an incremental running protocol [16].

The current study has some limitations, including the specific type of occlusal splints used and the two-dimensional kinematic analysis. Future research should explore a broader range of splint designs and their potential impacts on running mechanics by employing advanced motion analysis techniques. Additionally, exploring how these intraoral splints can be instrumented with sensors capable of detecting fatigue conditions through oral biomarkers is also a hypothesis to be considered in future research. Our findings enhance the understanding of progressive changes and the mandibular advancement effects in running patterns during a run to exhaustion at severe intensity. Practically, recognizing these adaptations is crucial for optimizing training regimens and reducing the risk of injury related to altered biomechanics under fatigue when running at or close to v$\overset{˙}{V}$O_2max_.

## 5. Conclusions

A biomechanical response to fatigue was observed during running until exhaustion at a severe intensity under both occlusal splint conditions, even though runners adopted different running approaches to compensate for underlying alterations. Complementarily, although no major kinematical splint effects were observed, runners appear to have implemented a running technique strategy that extended the distance covered during trials with the mandible advanced. Despite the intriguing finding of more balanced foot contact times when wearing an occlusal splint with mandibular advancement, when compared to its apparent impact on physiological variables, the use of these specific intraoral devices appears to have only a trivial contribution to kinematical changes when running until exhaustion at v$\overset{˙}{V}$O_2max_. Therefore, the increased running distance covered may hypothetically be primarily attributed to the previously reported physiological benefits of mandibular advancement devices rather than a direct impact on kinematics. Nevertheless, additional research is warranted to elucidate potential alterations in running patterns associated with the use of these specific occlusal splints.

## Data Availability

All data were contained within the manuscript.
